# ‘I decided to go back to work so I can afford to buy her formula’: a longitudinal mixed-methods study to explore how women in informal work balance the competing demands of infant feeding and working to provide for their family

**DOI:** 10.1186/s12889-020-09917-6

**Published:** 2020-12-02

**Authors:** Silondile Luthuli, Lyn Haskins, Sphindile Mapumulo, Nigel Rollins, Christiane Horwood

**Affiliations:** 1 Centre for Rural Health, University of KwaZulu-Natal, Durban, South Africa; 2 Department of Maternal, Newborn, Child and Adolescent Health, World Health Organization, Geneva, Switzerland

**Keywords:** Breastfeeding, Childcare, Workplace, Working women, Maternal health, Child health, South Africa, Africa

## Abstract

**Background:**

In South Africa almost 2 million women work informally. Informal work is characterised by poor job security, low earnings, and unsafe working conditions, with high rates of poverty and food insecurity. The peripartum period is a vulnerable time for many working women. This study explored how mothers navigate the tension between the need to work and the need to take care of a newborn baby, and how this affects their feeding plans and practices.

**Methods:**

A mixed methods longitudinal cohort method was employed. Informal workers were recruited in the last trimester of pregnancy during an antenatal visit at two clinics in Durban, South Africa. Data were collected using in-depth interviews and quantitative questionnaires at three time points: pre-delivery, post-delivery and after returning to work. Framework analysis was used to analyse qualitative data in NVIVO v12.4. Quantitative analysis used SPSSv26.

**Results:**

Twenty-four participants were enrolled and followed-up for a period of up to 1 year. Informal occupations included domestic work, home-based work, informal trading, and hairdressing, and most women earned <R3000 (US$175) per month. Participants had good knowledge of the importance of breastfeeding for child health. Most women planned to take time off work after the birth of their babies, supporting themselves during this time with the child support grant (CSG) received for older children, their savings, and support from the baby’s father and other family members. However, financial pressures forced many mothers to return to work earlier than planned, resulting in changes to infant feeding practices. Several mothers tried expressing breastmilk, but only one was able to sustain this while away from the baby. Most participants introduced formula, other foods and fluids to their babies when they returned to work or stopped breastfeeding entirely, but some were able to change their work or adapt their working hours to accommodate breastfeeding.

**Conclusions:**

Interventions are needed within the social and work environment to support mothers with breastfeeding while they continue earning an income in the informal economy. The extension of the CSG to the antenatal period could assist mothers to stay at home longer post-delivery to breastfeed their babies.

**Supplementary Information:**

The online version contains supplementary material available at 10.1186/s12889-020-09917-6.

## Background

The informal work environment provides paid work for millions of women globally [1], many of whom are also mothers of young children. Informal workers lack the social protection legislated for formal workers, including access to maternity leave, sick leave or unemployment benefits. Informal work is characterised by poor job security, low earnings, and unsafe working conditions, and there are high rates of poverty, food insecurity and vulnerability among informal workers [2, 3]. In South Africa among 6.8 million working women, almost 2 million women work informally, most commonly as informal traders, domestic workers and agricultural workers [4].

The peripartum period is a vulnerable time for many working women. The arrival of a new baby leads to increased financial pressure and substantial childcare responsibilities, at a time when women are often unable to work. Women with children usually take more responsibility for household and childcare work than men [5], are less able to seek and undertake paid work, and consistently earn less than men in similar positions [2]. This is particularly true for informal women workers, who are low paid and vulnerable to losing their jobs if they take prolonged leave. As a result, many women in informal work return to work soon after childbirth [3, 6]. Thus, the need to work and provide for their family has a major impact on how these women care for and feed their children [5].

Sustained and exclusive breastfeeding is essential for child health and development, and is the single most important intervention for reducing child morbidity and mortality [7]. Despite ongoing and strong messaging about the benefits of breastfeeding, a mother’s decision to breastfeed her baby depends on a set of complex socioeconomic, societal and individual factors [8]. It has been shown in many formal work settings that returning to the work environment limits exclusive breastfeeding practices and leads to early cessation of breastfeeding [9–11]. Women who return to work, including those who planned to breastfeed before childbirth, are more likely not to breastfeed or to breastfeed for a shorter period [10]. Breastfeeding is particularly important among economically deprived communities, including informal workers, because the high cost of breastmilk substitutes may mean that sustaining alternatives to breastfeeding is unaffordable [6], placing these babies at high risk of poor nutrition.

Little is known about infant feeding practices among women working in informal work settings, but evidence from the formal work environment suggests that the insecurity and vulnerability of informal workers is likely to result in early return to work and low rates of breastfeeding. A small study conducted in KwaZulu-Natal supports this finding, showing that although most informal women workers initiated breastfeeding, many women stopped breastfeeding on returning to informal work [3]. However, a contrasting view is that the flexible nature of informality may actually encourage breastfeeding and this has been suggested in several settings [5, 12, 13]. Thus, informal women workers, particularly own account workers, may be able to adjust their working conditions to allow them to breastfeed and care for their baby, for example taking their baby to work, working shorter or flexible hours or working from home [5]. In addition, informal employment may provide mothers with access to informal support networks that are unavailable to formal workers [13].

Given the large and increasing nature of informal work globally, and the vulnerability of women informal workers and their children, it is important to explore feeding and childcare choices among these mothers. This can inform interventions to provide additional support for mothers in informal work, for example support for them to stay at home longer after birth, or support for breastfeeding in the informal workplace. We conducted a longitudinal cohort study among women working in the informal work environment to prospectively explore the choices and decisions they made about caring for and feeding their baby from pregnancy until they had returned to work. In this article, we explore how mothers in informal work navigate the tension between the need to earn a living and the need to take care of their newborn baby, and how this effects the way they feed and care for their babies.

## Methods

We conducted a longitudinal mixed methods cohort study over a period of 1 year. The aim of the study was to explore the lives of informal workers focussing on their plans, experiences and practices about infant feeding and work during pregnancy, after delivery and on returning to work. A qualitative longitudinal design focusses on the stories of individual participants and allows researchers to capture critical moments and change processes as they occur over time [14]. Further, using mixed methods allows for collection of a variety of data to answer the research question. In particular, in a longitudinal qualitative study contemporaneous collection of quantitative data can provide a reliable context for the qualitative analysis, allowing for a more comprehensive understanding of the topic [15].

### Study site

The study was conducted in two urban townships in Durban, KwaZulu-Natal (KZN). Economic development within these townships has increased informal business development, with women occupying a substantial space in the informal sector. There are a high proportion of female-headed households (approximately 47 and 38% in the two sites), and high rates of unemployment and poverty in these areas. Access to basic services is poor, fewer than half of households have access to water inside the home and sanitation facilities connected to sewerage, and many people continue to live in informal housing [16]. All primary child carers in low income households are entitled to a government child support grant (CSG) of R420 (approx US$30) per month [17].

### Recruitment and sampling

Participants were recruited in the last trimester of pregnancy, enrolled in the study, and followed-up for a period of up to 1 year. In order to be eligible for participation, women had to be informal workers, aged 18 years or older, and between 32 and 38 weeks pregnant. Informal work was defined as having no work contract, and not contributing to the South Africa mandatory Unemployment Insurance Fund (UIF). Domestic workers were separately defined as women working in private households undertaking domestic and childcare work, regardless of whether they had a contract or paid UIF. Women working less than 3 days a week, who had been in informal work for less than 6 months or who planned to leave the area after delivery were excluded from the study. The sample size was set at 24 mothers, which was considered adequate to reach data saturation, allowing for some loss to follow up among study participants.

Recruitment was conducted at two peri-urban primary healthcare (PHC) clinics during women’s antenatal care (ANC) visits. A trained field worker checked for participant eligibility by screening all pregnant women attending the clinic, using a structured screening tool. Positively screened women entered a recruitment process that included two further meetings with a researcher, away from the clinic, to obtain informed consent and collect baseline data. To allow participants time to carefully consider their participation and withdraw from the study if they wished, potential participants were formally enrolled into the cohort and assigned a study number only once baseline data collection was completed.

### Data collection

**Fig. 1:**
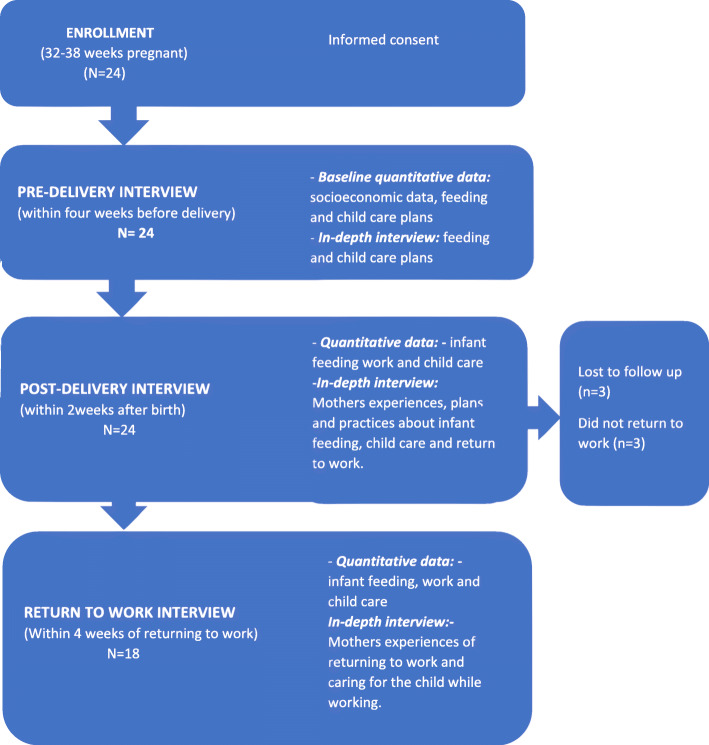
Participants and data collection

Participants were enrolled in the study during pregnancy and followed-up until the mother returned to work or the baby was 1 year old, whichever was the shorter period. Mothers were contacted by telephone every 2 weeks during the study period to track significant transition points for the mother and baby, and to schedule interviews. Interviews were conducted at a convenient place for the mother and were conducted in IsiZulu or English, according to the mother’s preference. Data were collected using quantitative questionnaires and in-depth interviews at three time points, pre-delivery, post-delivery, and after the return to work (Fig. 1).

### Quantitative data

We conducted a quantitative questionnaire at each time point. The pre-delivery questionnaire included mothers’ demographic information, father’s information, mothers work plans and feeding plans for after delivery and on returning to work (Supplementary file 1). The post-delivery questionnaire and the return to work questionnaire included information on current feeding practices as well as work practices and plans. Current feeding practices were determined using a 24-h food and fluid recall questionnaire (Supplementary file 2). Quantitative data were collected with an electronic tablet using proprietary software and uploaded to a centralised server in real time.

### Qualitative data

In-depth interview guides were designed to reflect the themes to be explored at each transition point, including relevant recurring themes and new topics specific to the recent transition. In-depth interviews explored participants’ plans and experiences of work and feeding at each time point. The interviews also explored the contrast between work and feeding plans during pregnancy, at post-delivery, and at return to work, and the mothers’ practices at each time point (Supplementary files 3, 4 and 5). The post-delivery interview was conducted within 2 weeks of delivery and the return to work interview within 4 weeks of returning to work (Fig. 1).

Data was collected by experienced qualitative researchers (SL, SM) using a semi-structured interview guide. The lead researcher (SL) has training in qualitative research to masters level and SM to honours level. Both researchers were female, were working for the affiliated institution during data collection, and had no relationship with the participants prior to enrolling in the cohort. Relationships were built over time with the participants through constant engagement allowing participants to be open during interviews and to speak about sensitive and personal issues that impacted on their own and their babies daily lives.

Interviews were between 15 and 60 min in length, and were conducted at the mother’s home in a private room away from the family or in the car when privacy was limited, according to the participants preference. All interviews were audio recorded.

### Data analysis

In-depth interviews were transcribed verbatim, translated to English, and quality controlled by researchers prior to data analysis. The study employed framework analysis using NVIVO v12.3 to analyse the data [18]. Framework analysis was chosen because it provides an established approach to ordering the data to reduce data volume and prioritize questions when working with a large dataset. Framework analysis provides coherence, structure and a systematic approach to analysis while retaining the thick description and complexity of the data. In addition, given the longitudinal nature of the study, this method allows between- and within-case comparisons [18].

At the start of the analysis process, three researchers (including SL and SM), independently familiarised themselves with the data and coded a sample of transcripts to identify the initial coding framework based on a priori themes derived from research questions [18]. A meeting between the coding researchers and the research team was held to discuss the themes to be included and to finalize the coding framework to be followed throughout the analysis process. The team met on a weekly basis to discuss the coding process and emerging themes.

Descriptive analysis using SPSS v26 was employed to analyse the quantitative data. Data is presented as frequencies only.

### Ethical considerations

Ethical approval was obtained from the University of KwaZulu-Natal Humanities and Social Sciences Research Ethics Committee (HSSREC) (HSS/0319/018), the KZN Department of Health (HRKM235/18) and the World Health Organisation Ethics Committee (ERC 0003101). All the participants provided written informed consent to participate in the study. Participants were reimbursed with R150 (US$8) for each interview to compensate them for time away from their work. To maintain anonymity and confidentiality, participants were allocated a study number that was used throughout the study period and names were not used. Participants were given assurance that no identifiable information will be linked to interview contributions.

## Results

Thirty-five eligible women were approached to participate, amongst whom 11 mothers failed to complete the recruitment process, either because they could not be contacted or because they refused to participate further. Twenty-four women were enrolled in the cohort.

This analysis relates to 18 mothers from the cohort who returned to informal work during the study period, three mothers who did not return to work and three who were lost to follow up were excluded from this analysis (Fig. 1). We report the results from 54 interviews, comprising 18 interviews conducted at each of three time points (pre-delivery, post-delivery and return to work), including both qualitative and quantitative data. Interviews were conducted between July 2018 and August 2019.

We describe the plans mothers made before delivery about feeding the baby and returning to work, and contrast these plans with the mothers’ practices after the baby was born, to explore the interaction between infant feeding, returning to work and the informal work environment.

### Demographic characteristics

The median age of participants at the pre-delivery interview was 28.5 years (sd 4.7; IQR 25.0–30.7). All women were still in a relationship with the baby’s father and many were living with him. Few women (5) had completed secondary school education. Women were working at various jobs including as own account (self-employed) workers, as employees in informal businesses, and as domestic workers. Most participants reported earning less than R3000 (approx. $175) per month (Table 1).

**Table 1:** Participants sociodemographic characteristics from baseline quantitative interview

Mothers	***N*** = 18
Population group (Black/African)	18
Relationship status	
Married	1
In a relationship and living with partner	11
In a relationship and not living with partner	6
Education	
Secondary schooling: grade 8 to grade 11	13
Completed schooling: grade 12	5
Number of children	
None (first pregnancy)	1
1–2	14
3–4	3
Pregnancy was planned	7
Self-reported HIV positive (all on antiretroviral treatment)	7
Receives financial support from father of baby	18
**Description of work**	
Own account worker (self-employed)	8
Employed in an informal business	4
Domestic worker	5
Informal employee in a formal business	1
**Type of work**	
Domestic worker	6
Hair dresser	5
Homebased worker (sewing, informal traders, beadwork)	4
Informal trader	1
Other (fuel attendant and informal tuck shop owner)	2
**No. of days worked per week**	
3–4 days	8
5–6 days	4
7 days	6
**Income per month**	
Less than R1000	3
R1000 – R3000	14
Above R3000	1

#### Pre-delivery plans

##### Pre-delivery work plans

Against the backdrop of informal work, low earnings and the need to prepare for the new baby, mothers made plans about how they would feed the baby, how much time to take off from work before the baby was born, and when to return to work after the birth.

A number of women (9) reported at the time of pre-delivery interview that they had stopped working ahead of the baby’s birth, and a further three mothers were planning to take time off work before the baby was born. The remaining six mothers were planning to continue to work up until the birth. All women planned to take some time off work after the baby was born. Most mothers planned to return to work before the baby was 2 months old, while some mothers planned to return to work when the baby was older (Table 2). The mother below describes how she will stay at home to continue breastfeeding.

**Table 2:** Planned and actual feeding practices and return to work (quantitative data)

***N*** = 18	Plans during pregnancy (Pre-delivery interview)	Practice after delivery (post-delivery interview)	Practice after return to work (return to work interview)
**Infant feeding practices**			
Initiated breastfeeding	–	16	–
Exclusive Breastfeeding	12	10	2
Formula milk only	4	4	8
Mixed-breastfeeding (breast and formula)	2	4	8
**Return to work (age of baby)**			
Less than 1 month	–	4^a^	1
1–2 months	10	–	9
3–4 months	6	–	3
5–6 months	2	–	4
Above 6 months	–	–	1

^a^includes three mothers who had not returned to previous work but did some paid work within2 weeks after delivery of baby

‘I will breastfeed her [baby], while I am still at home, maybe for about 4-6 months. I will return to work when she [baby] is six months old and I will feed her [baby] formula then’ (M13, hairdresser, pre-delivery interview)*.*

##### Financial support strategies and plans

Participants planned to use a variety of income sources to support themselves while they were not working, including their savings, the SA government child support grant (CSG) they received for their older children, and support from the baby’s father or from other family members. A few women (4) mentioned that they would continue receiving income from their employer or would continue working from home. Women also mentioned that they would apply for the CSG as soon as the baby was born.

‘I do not know because I will be forced to stay at home. I do not know how I will cope. I do not want to lie. The government also provides some assistance in its way. Maybe I will try and get a child support grant’ (M15, hairdresser, pre-delivery interview)*.*

##### Pre-delivery feeding plans

All mothers had attended antenatal clinic where breastfeeding was strongly encouraged by health workers, and before the baby was born most mothers planned to exclusively breastfeed (12) or to mixed breastfeed (2) their babies after birth.

‘I will give her [breast] milk. I will give her [breast] milk until she turns 2 years old. I will give her [breast] milk for the full period…In the morning I have to feed her before I leave. I will then express breast milk and leave it. I will have to reduce my working hours so that I can come back home early. I will keep track of time so that I can come back and breastfeed the baby because you can express the milk and leave it’ (M18, home-based worker/sells products, pre-delivery interview)*.*

A few women (4) planned to formula feed from birth, they mentioned that early return to work was the primary reason for choosing to formula feed their babies (Table 2).

‘We learned that we should breastfeed. I told her [nurse] that I will not be able to breastfeed because I will return to work soon’ (M07, domestic worker, pre-delivery interview)*.*

All mothers who planned to breastfeed their babies also planned to continue breastfeeding after returning to work. Mothers planned to either express breastmilk to feed the baby while she was at work or to introduce other foods and fluids to feed the baby during work hours and continue breastfeeding when at home. In addition, some mothers (4) planned to continue working at home in order to maintain breastfeeding for the period of 6 months. Mothers feeding plans are shown in Table 2.

#### Post-delivery experiences of feeding and work

At the post-delivery interview, 10 mothers reported that they were exclusively breastfeeding their babies as they had planned to do. However, some mothers had changed their feeding practices from their plans to exclusively breastfeed (Table 3). These mothers reported several reasons for changing the baby’s feeding practices within 2 weeks of the baby’s birth, including breastfeeding challenges such as mother’s perceptions that she did not have enough milk or that the baby was not satisfied, as well as a lack of knowledge regarding breastfeeding in the context of HIV. Family influences on feeding practices played a strong role, with older members of the family frequently advising mothers to add formula milk to the baby’s diet. In addition, a few mothers mentioned return to work as a key reason for changing feeding practices within 2 weeks post-delivery and some mothers wanted the baby to get used to formula milk before she returns to work.

‘I want her [baby] to get used to it [formula milk] so that when I am work there will not be any problem. I do not want her to focus on breastfeeding only’ (M21, domestic worker, post-delivery interview)*.*

**Table 3:** Participants plans and practices for feeding and returning to work

Mother characteristics (Baseline quantitative interview)	Pre-delivery Feeding plans and duration (baseline quantitative interview)	Feeding practice post-delivery (post-delivery quantitative interview)	Age of baby at return to work (return-to-work quantitative interview)	Feeding practice at return to work (quantitative data: return-to-work interview)	Details of child care at return to work: person and place (return-to-work quantitative interview)	Feeding and work balance (return-to-work qualitative interview)
M01 Age range: 20–24 years Domestic worker	Breast feed 3 months	Mixed breastfeeding Introduced formula milk at 7 days	6 days	Now breast feeding only (stopped giving formula milk)	Mother/non-relative (mother takes the baby to work/leaves at carer’s home)	“Granny did not know what to do because I had expressed the milk in the morning; they had to warm the milk for her and then give it to her; they failed to do that. They called me and I had to come back. I then went to the lady to continue [working] the next day”.
M05 Age range: 25–29 years Home based worker (sewing)	Breast feed 24 months	Exclusive breastfeeding	1 month	Exclusive breastfeeding	Mother (mother working from home)	“The change is that she does not get breastfed for the entire day anymore. She feeds from a bottle.”
M06 Age range: 20-24 years Hairdresser	Formula feed	Mixed breastfeeding	1 and a half months	Formula feeding	Other relative (mother working from home)	“Before I start doing client’s hair I make sure that I have prepared at least two bottles, you see. It is very rare to find that the bottles have been finished in an hour or two because she does not feed a lot. But if it happens, let us say she finished her bottle, granny knows how to prepare the formula for her”.
M07 Age range: 35–39 years Domestic worker	Breast feed 1 month	Formula feeding Never breastfed	6 weeks	Formula feeding	Other relative (Carer’s home)	“My sister feeds her. I give her formula. There is formula that is kept at my house and formula that is kept at my sister’s house”.
M08 Age range: 25–29 years Fuel attendant	Breast feed 1 month	Formula feeding Never breastfed	2 months	Formula feeding	Non-relative (Crèche)	“I wake up in the morning and prepare everything. I know how much she eats. Her carer also knows. I also pack an extra tin just in case. If she finishes these two bottles that I prepared for her, she can then make another one for her”.
M11 Age range: 25–29 years Tuck shop owner	Formula feed	Breastfeeding initiated Stopped at 5 days & introduced formula	1 month	Formula feeding	Mother (mother working from home)	“She is fed formula. I know it is not right. I also feed her Purity that is in the bottle…there are not too many challenges. Sometimes when she wants to sleep she cries. I have to soothe her while there are customers that want to buy. That is the main challenge”
M12 Age range: 35–39 years Domestic worker	Breast feed 12 months	Breastfeeding exclusively	3 months	Mixed breast feeding Introduced other food/fluids at 3 months	Mother (Takes baby to work)	“I started giving my baby porridge when I went back to work... I was not going to have enough time to breastfeed her, so that is why I just decided to give her porridge then give her breast milk and she will just sit afterwards and be fine. I feed her at work”.
M13 Age range: 25–29 years Hairdresser	Breast feed 6 months	Breastfeeding exclusively	6 months	Formula feeding, stopped breastfeeding at 6 months	Non-relative (Crèche)	“I wanted to breastfeed for a full 6 months. After that, I introduced her to formula so she can go to crèche…I have to wake up and bath her, feed her, prepare her bottles that she will take with to crèche, and I also bath and prepare to go to work. I first drop her off at crèche and then I go to work”.
M14 Age range: 35–39 years Domestic worker	Formula feed	Breast fed for 2 h Changed to formula feeding	3 months	Formula feeding	Child’s grandmother (Mother’s residence)	“My mother prepares food for her in the morning because she stays at home. I am usually busy preparing to go to work. My mother is the only person that is able to prepare formula for her in the morning”.
M15 Age range: 20–24 years Hairdresser	Mixed breastfeeding	Mixed breastfeeding	1 month	Mixed breast feeding	Mother (Takes baby to work)	“She cries a lot. I ended up putting her on my back and going with her to work. So I worked with her on my back the whole time until I knocked off. It was extremely tough, you see, because you are working and the baby is on your back and does not want to be carried by anyone [but you] and she is crying. It was like that. Even now, when I go to work I take her with me because the problem is that the breast milk is on me so I cannot leave her”.
M16 Age range: 30–34 years Home based worker	Breast feed 6 months	Exclusive breastfeeding	8 months	Mixed breast feeding Introduced other food/fluids at 6 months	Mother (mother working from home)	“I have to do my work and I sometimes think I will do my work at night but then she just wakes up and wants to be close to me or even be breastfed you see. She suckles the milk so I have to make sure that I work hard when she is asleep because when she is awake I have to play with her and give her attention”.
M17 Age range: 25-29 years Domestic worker	Formula feed	Breastfeeding exclusively	5 months	Mixed breast feeding Introduced other food/fluids at 2 months	Non-relative (Crèche)	“I just wake up in the morning and make porridge for my baby. I give him porridge and if it finished I will make another porridge. And then I take out things for him to eat in crèche and bath him then I go and leave him in crèche”.
M18 Age range: 30–34 years Homebased worker, sells products	Breast feed 24 months	Mixed breastfeeding	4 months	Mixed breast feeding Introduced other food/fluids at 3 months	Child’s grandmother (Mother’s residence)	“I make sure that I feed her before I leave. She eats (commercial baby food). She eats and drinks milk and I have introduced her to formula and she has gotten used to it…I leave her with formula and when I come back I breastfeed her”
M20 Age range: 25–29 years Hairdresser	Breast feed 6 months	Mixed breastfeeding	1 month	Formula feeding	Mother (Residence – mother working from home)	“I tell the person beforehand if they want me to do their hair that I have a child so if I have to go and feed the child they must bear with me. It is up to them. If they will not tolerate me feeding my child then they can go to another hair stylist”.
M21 Age range: 25–29 years Domestic worker	Breast feed and other foods 24 months	Mixed breastfeeding	3 months	Mixed breast feeding	Non-relative/ crèche	“She eats in the morning before I go to work. I make porridge and feed her and also put some in a lunch box. I then prepare her bottle and also express breast milk … I express it and put the bottle in a flask”.
M22 Age range 30–34 years Hairdresser	Breast feed 6 months	Mixed breastfeeding	2 months	Formula feeding	Other relative (Mother’s residence)	“Whenever I called to check on her they said she was fine and she was feeding on the milk that I had bought her. They said it was not affecting her”.
M23 Age range: 30–34 years Homebased worker/informal trader	Breast feed 3 months	Exclusive Breastfeeding	6 months	Mixed breast feeding Introduced other food/fluid at 2 months	Child’s father (Mother’s residence)	“I used to keep time every day and ensured that I leave the house at the same time every day. Now when I leave, sometimes the child is already awake, so I have to first breastfeed him before I leave”.
M24 Age range: 25–29 years Market trader (works for employer)	Breast feed 6 months	Breastfeeding (plus water)	2 months	Mixed breast feeding Introduced other food/fluid at 2 months	Non-relative (Crèche)	“When I wake up in the morning I prepare a bottle for her to feed on quickly. I then take her formula and put it in her bag… I then give all these things to the lady that is caring for her so that as soon as the child finishes her bottle the carer can prepare another bottle and keep boiled water in the flask and use it to prepare the bottle for the child…She is breastfed when I come home in the evening. She is also breastfed at night”.

#### Return to work

##### Mothers who returned to work earlier than planned

Four mothers had already started working or had done some paid work within 2 weeks of the baby being born, returning to work earlier than planned. This included one mother who returned to her previous work and three mothers who took on casual paid work or adapted their work and work environment during this time to supplement their income, before returning to their previous job later.

‘No, I have not gone back to work. I just pop in to check if everything is going well’ (M11, tuck shop owner, post-delivery interview)*.*

Reasons for returning to work soon after delivery were primarily financial pressures, including having to buy baby formula because the baby was hungry. One mother who was losing weight was advised by family members to stop breastfeeding and to give the baby formula.

‘She is breastfed but I was advised at home to stop breastfeeding because I was losing a lot of weight. They advised me to buy her Infacare [infant formula] instead. So, I went to do the laundry and got R100 which I used to buy the Infacare with’ (M01, domestic worker, post-delivery interview)*.*

In addition, two mothers who returned to work within 2 weeks of delivery were able to do so because they were working from home or were able to adapt their work so that they worked from home. One mother ran a tuckshop from home, and although she had employed someone to help her, she opted to work part-time to monitor income from the tuckshop daily. Another mother who had previously sold goods outside the school premises was able to adapt her work so that she was selling her goods at home after the baby was born because she had no other source of income.

‘I took a break [from selling at the school]…I was still selling from the house as I told you’ (M23, home-based worker/informal trader, post-delivery interview)*.*

Half of the mothers (9) returned to work when the baby was aged between one and 2 months, for six of these mothers this was earlier than planned. The main reason for early return was financial pressures such as savings being exhausted or not having money to cover household expenses and/or needs of the baby (nappies, clothing, and formula milk).

‘I went back to work because my baby was starving and it seemed like breast milk alone was not enough to satisfy her. So, I decided to go back to work so I can afford to buy her formula. So I bought it. I also bought stuff that I needed for myself. So, I was also struggling and then I decided that it will be better if I return to work instead of suffering’ (M24, market trader, return-to-work interview)*.*

Three mothers were able to return to work within three to four months as they had planned. These mothers received financial support from the father of the baby, family members, and had savings to take care of the baby during this time.

Five mothers returned to work between 5-8 months. One mother who had intended to return to work when the baby was 6 months to exclusively breastfeed her baby for the full 6 months was able to keep to her plan.

‘What made me wait 6 months is that my baby was being breastfed. I wanted to breastfeed for a full 6 months’ (M13, Hairdresser, return-to-work interview)*.*

##### Mothers who returned to work later than planned

In contrast to most mothers who had to return to work earlier than planned, four mothers had to delay returning to work because of poor health or challenges with feeding the baby, leading to severe financial hardships for these mothers. For example, one mother was unable to return to work because the baby refused any food other than breastmilk. This mother relied on support from the father of the baby, the CSG for her older children, and income from her children selling items at school.

‘I am able to buy a few things when I have gone to get my child support grant. I buy things such as lollipops and chips and my children do sell those at school because it is allowed…that helps me a lot to perhaps have a little bit of money; if I am short of something I am able to use that money’ (M16, home-based worker, return-to- work interview)*.*

#### Feeding practices after returning to work

Participants adopted different feeding practices when they had returned to work, depending on the type of work and the number of hours worked. Mothers who left their baby in non-parental care during working hours (11), either stopped breastfeeding, introduced other foods and fluids while continuing to breastfeed, or maintained breastfeeding by feeding the baby expressed breastmilk during working hours. Mothers who were home-based workers (4) or took the baby to work (3) were able to breastfeed their babies during working hours (Table 3).

##### Expressing breastmilk

Eight mothers reported that they had tried to express breastmilk to give to the baby when they returned to work. However, only a few mothers (4) reported they were able to express breastmilk more than 10 times over 2 weeks, and only one mother was able to maintain exclusive breastfeeding by expressing breastmilk after returning to work.

‘If I am around like in the morning and when I come back [from school] in the evening I breastfeed her but during the day when I am at work or at school I leave her with expressed milk. I express the milk and they give it to her while I am gone’ (M05, home-based worker, return-to-work interview)*.*

Other mothers reported that they were unable to express enough milk for the baby to feed on while at work leading mothers to add other foods and fluid.

‘Initially I had said I would express breast milk for her but she drinks a lot. So now she drinks [formula milk]. If I leave her with expressed milk she does not get full and it runs out quickly. So, I leave her with formula and when I come back I breastfeed her’ (M18, home-based worker/sells products, return-to-work interview)*.*

Another mother had difficulty in expressing breastmilk leading to serious problems because she could not afford to buy formula milk, so the baby was fed with warm sugar water when she is at work.

‘When I am away this little man does not drink any milk. He is given a spoon of warm sugar water. The same applies if I am gone to order stock. That is what he does. It is not good practice but it is better than him not drinking anything at all. He gets thirsty because he now eats food. Once he has eaten and had something to drink, but he still gets thirsty. He [father] calls me and tell me that the baby is thirsty so I must try to hurry back home’ (M23, home-based worker/informal trader, return to-work interview)*.*

Home-based working mothers were able to breastfeed but still had to leave the baby at times, such as when they went to purchase stock, and feeding the baby at this time was a challenge. Some mothers tried expressing breastmilk but they found it difficult, and their babies struggled to feed from a bottle.

‘When I tried to feed her with a bottle she would bite the nozzle and that milk would get splashed on her neck. She was not swallowing it so I saw that I was just wasting my time. I ignored her again and tried to give her the bottle at night but she refused to drink it; she would start touching me trying to find my breast. I did not succeed in doing that’ (M16, home-based worker, return-to-work interview)*.*

##### Mixed breastfeeding

Among mothers who left their baby in the care of others on returning to work, some mothers (5) mixed breastfed giving both breastmilk and other food and fluids. Mothers left maize meal porridge, formula milk, expressed breastmilk and commercial baby food for the baby to eat during working hours and mothers continued to breastfeed after work. Other food and fluids were added as early as in the first 1 month (Table 3).

##### Stopped breastfeeding

Six mothers had stopped breastfeeding and were feeding the baby formula milk and other foods and fluids at the return-to-work interview. Returning to work and being away from the baby were key reasons mothers stopped breastfeeding their babies. It was common practice for mothers to change feeding practice a week prior to returning to work to monitor how the change in feeding practices affected the baby.

‘It has been almost a week now. I weaned her off on [date] before I started working. It was a Wednesday. I bought [formula milk] and introduced her to it…I fed her the other milk because I wanted to see how she would react to it. If it was making her have a runny tummy I was going to stop giving it to her and try another brand. It did not give her a runny tummy so I continued to feed her. Everything is going well so far’ (M22, shop assistant, return-to-work interview)*.*

#### Mothers who cared for the baby themselves

##### Taking baby to work

Three mothers took the baby with them to the workplace and were able to care for the baby themselves and maintain breastfeeding. They chose to take the baby to work with them because they did not trust crèches or childcare facilities but preferred to take care of the baby themselves. However, balancing work and breastfeeding was difficult and required changes to the mothers’ work hours and workload to allow for feeding time. One mother arranged with her employer to start work early, missed her tea and lunch breaks, and worked late to cover the time used for feeding the baby during working hours. Another mother reported that clients and co-workers assisted her to allow time for feeding during working hours. However, she was unable to attend to many clients in a day so her income was reduced.

‘There are changes. I now start work at half past 7 whereas in the past I used to start at 8 and I leave work late just like yesterday… they called me in and I did go to work. I started at half past 7; I got there at half past 7 and left at half past 4; yes. The reason for that is because as you are working you also have to take care of the baby. As you are working the baby also wants to be fed so you will sit down and feeding the baby will take time. So, you have to continue working; you just cannot leave when you have not completed working’ (M12, domestic worker, return to work interview)*.*

##### Mothers who worked from home

Working from home was an option that allowed mothers to feed and care for the baby themselves. However, balancing work and breastfeeding while working from home was difficult. The four mothers who worked from home said they had to ensure that the baby was fed and asleep before starting work. When their babies woke up, the mother often stopped work to take care of the baby’s needs. This had an impact on the work with mothers often not meeting work targets and the baby feeding was disrupted.

‘There is change because I can no longer work for the whole day or full time. I only do my work when I have time, maybe when the baby is sleeping. I try to work quickly. But when the baby wakes up I cannot continue working. So, I stop and the work piles up’ (M05, home-based worker, return to work interview)*.*

## Discussion

Our study showed that most pregnant informal workers planned to breastfeed their babies and stay at home, at least for a short period. However, challenges experienced after delivery led many mothers to change their plans, with the result that breastfeeding duration was shorter, other foods were added to the baby’s diet, or breastfeeding was abandoned altogether. Like all new mothers, many women in informal work experienced challenges to initiating and sustaining breastfeeding in the first few weeks of the baby’s life [19, 20]. Informal workers also had much in common with formal workers, so that returning to work or the prospect of returning to work reduced the rate of breastfeeding initiation and shortened the duration of breastfeeding [9–11, 21]. In addition, mothers in informal work faced further challenges as a result of the vulnerability, low pay and job insecurity that are typical of informal work. Increasing financial pressures when the baby was born and concerns about keeping their job meant these mothers had to make difficult choices about how to balance plans to breastfeed with the need to work and support their family. For some mothers these challenges led to a vicious cycle, whereby adding formula milk to the baby’s diet increased the financial pressure on women, leading to early return to work.

Breastfeeding has substantial and lifelong positive health benefits for both mother and baby, and is important for the baby’s cognitive development [6, 7]. High levels of poverty and food insecurity among informal workers [3], put their children at high risk of malnutrition and failure to reach their long-term developmental potential. Therefore, in the context of informal work, breastfeeding is particularly crucial to prevent the cycle of intergenerational poverty, where malnutrition is a key driver. To successfully breastfeed, women require support at home and in the workplace, and financial support to stay at home and breastfeed the baby.

### Financial support

Our study clearly showed that financial pressure to provide for the family and the new baby were the most important factors driving mothers’ decision-making, often forcing them to return to work early. While away from work, mothers relied on financial support from their family and the baby’s father and the CSG received for older children, but this support was frequently insufficient even for their basic needs. It is well accepted in formal work settings that longer durations of paid maternity leave have resulted in increased initiation and duration of breastfeeding [10, 22, 23]. Given the important benefits of breastfeeding for the vulnerable children of informal workers, governments should consider investing in the future of these children by providing maternity grants to women in the informal work environment. In South Africa, the CSG provided by the Department of Social Development has been shown to alleviate poverty and hunger among children in low income families [24, 25], and this grant could be extended to include the antenatal period. Financial support before and after delivery would assist women in informal work to stay at home longer to breastfeed their babies, with long term benefits for the health and development of their children. In addition, increased breastfeeding duration would lead to substantial savings for the health system, as a result of improved child morbidity and mortality [8].

### Workplace support

In the formal workplace it has been clearly shown that a supportive work environment can improve breastfeeding duration [26]. A range of workplace interventions are effective in improving breastfeeding, including providing mothers with information and guidance about breastfeeding while working, providing facilities and policies to support breastfeeding in the workplace, for example facilities for hygienic breastmilk storage, flexible hours or breastfeeding breaks [27]. Support from employers and co-workers, even without additional facilities, can also improve duration of breastfeeding for working mothers [28]. We believe that similar strategies could be implemented within the informal work environment to assist mothers to continue breastfeeding.

Women working informally may have the advantage of working flexible hours with the option of working with the child or working from home, so that the informal work environment may appear supportive for breastfeeding. However, many informal workers work in unsafe, unhygienic, unsupportive, and vulnerable working conditions, which has an impact on their decision to breastfeed after returning to work [3]. In addition, for many women bringing their children to work with them, breastfeeding in public spaces is deemed as culturally unacceptable, making it difficult to continue with breastfeeding [6]. Interventions in the informal work environment should include private spaces for childcare and breastfeeding for mothers. Moreover, there is a need for education and awareness on the importance of breastfeeding within the work environment to increase the level of support among colleagues, and this could be extended to community and family level.

### Health system support

Another important finding from our study was that many participants had already been unable to successfully implement their plans to exclusively breastfeed, even before returning to work. Mothers in informal work experienced the same breastfeeding challenges that are common among all breastfeeding women [19], in particular the perception that breastmilk is insufficient for the babies needs and the baby requires additional foods. Participants reported receiving information about infant feeding during visits to the ANC, but lacked support from the healthcare services to maintain breastfeeding post-delivery, highlighting the importance of breastfeeding support for all women. The health system at all levels, including community health workers (CHWs) working at household level, has a strong role to play in supporting breastfeeding, particularly to support sustaining breastfeeding in the early weeks of the baby’s life when most breastfeeding challenges are experienced.

In addition, the health system has a particular role to play to support working mothers to continue breastfeeding after returning to work. Most of our participants planned to exclusively breastfeed their babies for 6 months, and continue breastfeeding after returning to work, but only one mother succeeded in achieving her plan. Expressing breastmilk to maintain breastfeeding while away from the baby requires skills and commitment from the mothers, and support from health workers and the community. Health workers should identify working mothers from among those mothers attending postnatal health services, and give counselling tailored to the needs of mothers planning to return to work. CHWs could also play a significant role in supporting informal workers with maintaining breastfeeding while working. For many mothers the informal workplace is a public area, and CHWs could play a role in supporting mothers breastfeeding in the workplace, for example by visiting local workplaces to provide health education to co-workers and encourage supportive measures for breastfeeding in the workplace.

### Community support

Social support systems play a crucial role in providing mothers with support for infant feeding decisions and for continuing with breastfeeding [20]. The father of the baby, family members, clients, and neighbours were key support systems mentioned by mothers in our study, supporting them with childcare and feeding. Some of the support systems were also mentioned as key influencers on decisions about infant feeding. Interventions aimed at educating and upskilling these support structures with knowledge on the importance of breastfeeding and skills to assist mothers with breastfeeding could help in increasing duration of breastfeeding among these mothers.

### Strengths and weaknesses of the study

This study employed a strong longitudinal mixed-methods design, which allowed us to develop an in-depth understanding of the lives of informally working mothers and their experiences with balancing informal work and infant feeding. Collecting data prospectively over an extended period provided opportunities for researchers and mothers to build relationships which allowed mothers to discuss sensitive issues, and researchers were able to gather data about events as they happened. This provided high quality, rich data to develop in-depth insights into the topic, but the design does not allow the findings to be generalised to the wider population of informal workers.

## Conclusion

Our study showed that for mothers working in the informal work environment, financial pressure to provide for their household and family was the key factor that undermined their plans to breastfeed and spend time at home with their new baby. Multi-sectoral interventions are required to support breastfeeding in this vulnerable group of women, including in the health system, in the community and in the workplace. We advocate for maternity grants for informal workers, to allow these mothers to spend more time with their babies and enable longer durations of breastfeeding to protect the health of these women and their families.

## Supplementary Information

**Additional file 1.** The Livelihood and Nurturing Care study (LiNCs): Baseline questionnaire.

**Additional file 2.** The Livelihood and Nurturing Care study (LiNCs): Follow-up questionnaire.

**Additional file 3.** The Livelihood and Nurturing Care study (LiNCs): IDI interview guide plans for feeding and child care (pregnancy).

**Additional file 4.** The Livelihood and Nurturing Care study (LiNCs): IDI interview guide plans for feeding and child care (post-delivery).

**Additional file 5.** The Livelihood and Nurturing Care study (LiNCs): IDI interview guide plans for return to work.

## Data Availability

All data, transcripts and study tools to support the findings of this study are available from the Centre for Rural Health and will be made available upon reasonable request from the principal investigator or corresponding author.

## Abbreviations

ANC: Antenatal care
CHW: Community health workers
CSG: Child support grant
PHC: Primary healthcare
UIF: Unemployment insurance fund
