# Supplementary material for: ‘I decided to go back to work so I can afford to buy her formula’: a longitudinal mixed-methods study to explore how women in informal work balance the competing demands of infant feeding and working to provide for their family
Source: BMC Public Health. 2020 Dec 2;20:1847. doi: 10.1186/s12889-020-09917-6 (PMC7709310; doi:10.1186/s12889-020-09917-6)
Supplement: Supplementary file 2 — Additional file 2. The Livelihood and Nurturing Care study (LiNCs): Follow-up questionnaire. [file 12889_2020_9917_MOESM2_ESM.pdf]

Follow-up questionnaire

Section 1: Administration

Section 1: Administration and Informed consent									
A1.1	Site of recruitment<br>**Indawo la kuxoxisanwa khona**								
A1.2	Tracking number<br>**Inombolo yokulandelela**								
A1.3	Interviewers name / initials<br>**Igama lomphenyi mibuzo/ Inishiyali**								
A1.4	Date of interview<br>**Usuku lwenxoxo mibuzo**	D	D	M	M	Y	Y	Y	Y
A1.5	Is this the first visit after your baby was born?<br>**Ingabe uyaqalwa ukuvakashelwa kusukela umntwana wakho ezaliwe?**	1= Yes				0= No → ***Skip to 2.5***			

Section 2: Feeding practices

**To be asked of all mothers regardless of which field visit this is**<br>**Kuzobuzwa kubobonke omama , noma ingabe ukuphi ukuvakashela**			
FP2.1	How old is your baby now?<br>**Ingabe mudala kangakanani umntwana wakho**	***Write date of birth***	
**I am going to talk about how you are feeding your baby now**<br>**Manje ngizoxoxisana nawe mayelana nendlela omupha ukudla ngayo umntwana wakho**			
FP2.2	Where was your baby Born?<br>**Wazalelwa kuphi Umntwana?**	1= Outside a health facility<br>2= In health facility	
FP2.3	What type of delivery did you have?<br>**Ingabe wamuteta ngaluphi uhlobo lokuteta umntwana?**	1= Vaginal delivery<br>2= Caesarean section	
FP2.4	How much did the baby weigh when he/she was born?<br>**Sasithini isisndo somntwana ngesikhathi ezalwa?**	………………………………<br>**N/A if you do not know**	
FP2.5	Have you ever breastfed your baby since he/she was born?<br>**Ingabe wake wamuncelisa ubisi lwebele umntwana wakho kusukelwa ezelwe?**	1= Yes	0= No<br>***Skip to 2.8***
FP2.6	Are you still breastfeeding your baby?<br>**Ingabe usamuncelisa umntwana wakho?**	1= Yes<br>***Skip to 2.8***	0= No
FP2.7	For how long did you breastfed?<br>**Wamuncelisa isikhathi esingakanani?**	…………………..<br>***(days, weeks or month)***	

FP2.8	How are you currently feeding your baby?<br>**Ingabe umupha kanjani ukudla okwamanje?**	1 = Breastfeeding only<br>2 = Formula feeding (no breast milk) ➔ ***Skip to 2.10***<br>3 = Mixed feeding (breast milk and other food) ➔ ***Skip to 2.10***	
FP2.9	Have you **ever** given any foods or fluids other than breast milk to your baby?<br>**Ingabe usuke wamupha umntwana wakho okunye ukudla noma okuwuketshezi ngaphandle kobisi lwebele?**	1 = Yes	0 = No<br>➔ ***Skip to 2.11***
FP2.10	At what age did you start to give (name) other foods or fluids other than breastmilk?<br>**Wayengakanani (igama) ngenkathi uqala ukumupha okunye ukudla noma okusaketshezi ngaphandele kobisi lwebele?**	1=……………………..Age<br>***(days, weeks or month)***	2=N/A<br>Write 99999
FP2.11	Have you ever given any water to your baby?<br>**Ingabe usuke wabupha umntwana wakho amanzi?**	1= Yes	0 = No
FP2.12	Have you ever given any "muthi" or traditional medicines (obtained from a traditional healer) to your baby?<br>**Ingabe umntwana wakho usuke wamupha noma imuphi umuthi noma imithi yesintu (oyithole kubalaphi bendabuko)?**	1 = Yes	0 = No
FP2.13	Have you ever given any formula to your baby?<br>**Ingabe usuke wamupha umntwana wakho ubisi lwefomula?**	1 = Yes	0 = No
FP2.14	Have you **ever** expressed breastmilk to give to your baby?<br><br>**Ingabe usuke walikhama ubisi lwebele ukuze umuphe lona umntwana wakho?**	1 = Yes	0 = No ➔ ***Skip to 3.1***
FP2.15	How often have you expressed breastmilk to give to your baby?<br><br>**Usuke walikhama kangaki ubisi lwebele ngenhloso yokupha umntwana wakho?**	1 = Rarely (one to two times in the past two weeks)<br>2 = Sometimes (three to 10 times in the past two weeks)<br>3 = Often (more than 10 times in the past two weeks)	

Section 3: 24 hour food and fluid recall

**TO BE ASKED FROM ALL MOTHERS – BREASTFEEDING, MIXED FEEDING AND FORMULA FEEDING MOTHERS**<br><br>I would like to ask you particularly about any liquids and semi-solid / solid food (with a spoon) that (name) may have had ***yesterday during the day and night (24 hours).*** I am interested in whether (name) had the item even if it was combined with other food.<br><br>I will start with liquids.<br><br>**Ngicela ukukubuza ngokusaketshezi noma ukudla okuthambile okudliwe umntwana (ngokhezo) u (igama) izolo emini noma ebusuku (emahoreni awu – 24). Ngifuna ukwazi ukuthi u (igama) ukutholile lokhu yize bekuxutshwe nokunye ukudla.**<br><br>**Ngizoqala ngakungamanzi**			
FR3.1	Did (name) drink plain water yesterday during the day or night?<br><br>**Ngabe u(gama) uke waphuza amanzi engaxutshwe nalutho emini noma ebusuku bayizolo?**	1 = Yes	0 = No
FR3.2	Did (name) drink infant formula yesterday during the day or night?<br><br>**Ngabe u(gama) uke waphuza ifomula/ubisi lwethini emini noma ebusuku bayizolo?**	1 = Yes	0 = No
FR3.3	Did (name) drink juice or juice drinks or tea (e.g. rooibos) yesterday during the day or night?<br><br>**Ngabe u(gama) uke waphuza ijusi noma itiye (isb. iroyibhosi) emini noma ebusuku bayizolo?**	1 = Yes	0 = No
FR3.4	Did (name) drink clear broth/clear soup yesterday during the day or night?<br><br>**Ngabe u(gama) uke waphuza umhluzi/isobho emini noma ebusuku bayizolo?**	1 = Yes	0 = No
FR3.5	Did (name) drink or eat vitamin or mineral supplements or ORS or any medicines *obtained from the clinic* or a doctor yesterday during the day or night?<br><br>**Ngabe u(gama) uke waphuza amavithamini noma amaminerali noma i- ORS noma imithi *oyithole emtholampilo* noma kudokotela emini noma ebusuku bayizolo?**	1 = Yes	0 = No
FR3.6	Did (name) drink or eat any muthi e.g. muthi nyoni,or traditional medicines *obtained from a traditional healer*, or bought over the counter yesterday during the day or night?	1 = Yes	0 = No

	**Ngabe u(gama) uke waphuza noma wadla imithi isb. Umuthi wenyoni, noma imithi yendabuko *etholwe kumlaphi wendabuko* noma ethengwe ekhemisi emini noma ebusuku bayizolo?**		
FR3.7	Did (name) drink or eat yogurt or amaasi or thin porridge yesterday during the day or night?<br>**Ngabe u(gama) uke waphuza noma wadla iyogathi, amasi noma**<br>**iphalishi elimanzi emini noma ebusuku bayizolo?**	1 = Yes	0 = No
FR3.8	Did (name) drink any other fluids/liquids yesterday during the day or night that I have not mentioned?<br>**Ingabe u(igama) usuke waphuza noma ihlobo yini yokusaketshezi izolo emini noma ebusuku engingakakusho?**	1 = Yes	0 = No➔ ***Skip to 3.10***
FR3.9	What other fluids/liquids did (name) drink?<br>**Iluphi olunye uketshezi u (igama) aluphuzile?**		
Now I will ask you about solid or semi-solid mushy food.<br>**Manje ngizokubuza ngokudla okuqinile noma okuthambile.**			
FR3.10	FR3.11 Did (name) have any other solid or semi-solid food that I have not mentioned during the day or night?<br>Ingabe u (igama) useke wakudla ukudla okuqinile noma okuthambile engingakubalanga emini noma ebusuku?	1 = Yes	0 = No<br>➔ ***Skip to 4.1***
FR3.11	What solid or semi-solid food did (name) have?<br>Tick all that are mentioned<br>**Ikuphi okunye ukudla okuqinile noma okuthambile akudlile u (igama)?**<br>**Beka uphawu kukho konke okushiwo**	1= Baby Cereal<br>2= Any yogurt, sour milk, amasi etc.<br>3= Commercial baby food (e.g. purity)<br>4 = Bread<br>5 = Porridge made with grains, Maize meal or oats<br>6 = Fruit<br>7 = Vegetables<br>8 = Meat<br>9 = Eggs<br>10 = Fish<br>11 = Cheese<br>12 = Sugary foods or sweets	

		13 = Commercial snack like chips or crisps etc
		14=Beans/lentils/seeds/samp
		15= Oil fats butter/ margarine
		16= Other
FR3.12	Specify if other selected or make notes:<br>**Cacisa noma ubhale kabanzi uma kukhethwe okunye**	

Section 4: Child health

I would like to ask you about any health problems your baby has had since the baby was born<br><br>**Ngicela ukukubuza mayelana nezinkinga eziphathelene nempilo yomntwana wakho, abenazo kusukela ezelwe**			
CH4.1	In the last 4 weeks has your baby suffered from any illness?<br><br>**Kulamasonto amane adlule, useke wagula umntwana wakho?**	1 = Yes	0 = No<br><br>➔ ***Skip to 4.4***
CH4.2	Where did you seek help for your baby when your baby was ill?<br><br>**Ingabe ngenkathi egula umntwana wakho, walufuna kuphi usizo?**	1= Managed child at home	
		2= Clinic or health facility	
		3= Chemist	
		4= Traditional healer	
		5 = Private doctor	
		6 = Other	
CH4.3	Specify if other selected or make notes<br>**Cacisa noma ubhale kabanzi uma kukhethwe - okunye**		
CH4.4	In the last 4 weeks, have you given your baby any medicines?<br><br>**Emasontweni amane ingabe umunikeza imithi noma eyanhloboni?**	1 = Yes	0 = No<br><br>➔ ***Skip to 4.6***
CH4.5	If yes, what medicines are you giving your baby?<br><br>**Uma kunjalo, inhloboni yemithi ozomunikeza yona?**		

CH4.6	Since your baby was born has your baby been admitted to the hospital?<br><br>**Selokhu ezelwe umntwana wakho, useke walaliswa esibhedlela ngoba egula?**	1 = Yes	0 = No<br><br>➔ ***Skip to 4.9***
CH4.7	How many times has your baby been admitted to hospital since birth?<br><br>**Uselaliswe izinkathi ezingaki esibhedlela kusukela ezelwe?**	……………………. times	
CH4.8	Why was your baby admitted to hospital (specify)<br><br>**Ingabe wayelaliselwe ini umntwana wakho?**<br><br>***(Most recent admission)***	…………………………………	
CH4.9	When your baby last went to the clinic who took your baby to the clinic<br><br>**Ngesikhathi u(gama) egcina ukuya emtholampilo wahanjiswa ubani?**	1= Myself (not during work time)<br>2= Myself (took time off work)<br>3= Family member<br>4= Non family member	
CH4.10	Have you been visited by a CCG in your home since birth?<br><br>**Kulenyanga edlule, ingabe usuke wavakashelwa u Nompilo ekhaya lakho?**	1 = Yes	0 = No<br><br>➔ ***Skip to 4.12***
CH4.11	How many times has the CCG visited you in your home in the last month?<br><br>**Kulenyanga edlule, ukuvakashele kangaki uNompilo ekhaya lakho?**	…………………………………….	
CH4.12	Do you receive child support grant for this baby?<br>**Ingabe uyayihola imali ye-grant ngalomntwana lo?**	1= Yes	0= No

Section 5: Food Security (USAID Household Food Insecurity Access Scale)

I would now like to ask you about the food you have available to feed yourself and the rest of your family.<br><br>**Ngithanda ukukubuza mayelana nokudla onakho ukuba uzondle wena kanye nabomndeni wakho.**		
FS5.1	In the past four weeks, did you worry that your household would not have enough food?<br><br>**Kulamasonto amane adlulile, uke wakhathazeka ukuthi akukho ukudla okwanele ekhaya lakho?**	1 = Rarely (one to two times in the past four weeks)<br>2 = Sometimes (three to 10 times in the past four weeks)<br>3 = Often (more than 10 times in the past four weeks)
FS5.2	In the past four weeks, were you not able to eat the kinds of foods you preferred because of a lack of resources?<br><br>**Kulamasonto amane adlulile, ngabe wena awukwazanga ukudla izinhlobo zokudla ozithandayo ngenxa yokungabi nazo izinsiza?**	1 = Rarely (one to two times in the past four weeks)<br>2 = Sometimes (three to 10 times in the past four weeks)<br>3 = Often (more than 10 times in the past four weeks)
FS5.3	In the past four weeks, did you have to eat a limited variety of foods due to a lack of resources?<br><br>**Kulamasonto amane adlulile, ngabe wena udle ukudla okushoda ngezinhlobonhlobo zokudla ngenxa yokungabi nezinsiza?**	1 = Rarely (one to two times in the past four weeks)<br>2 = Sometimes (three to 10 times in the past four weeks)<br>3 = Often (more than 10 times in the past four weeks)
FS5.4	In the past four weeks, did you have to eat some foods that you really did not want to eat because of a lack of resources to obtain other types of food?<br><br>**Kulamasonto amane adlulule, ingabe wena uye waphoqeleka ukudla ukudla ongakufuni ngenxa yokungabi nezinsiza zokuthola ezinye izinhlbo zokudla?**	1 = Rarely (one to two times in the past four weeks)<br>2 = Sometimes (three to 10 times in the past four weeks)<br>3 = Often (more than 10 times in the past four weeks)
FS5.5	In the past four weeks, did you have to eat a smaller meal than you felt you needed because there was not enough food?<br><br>**Kulamasonto amane adlulule, ingabe kwaphoqa ukuthi udle ukudla okuncane (epuletini/endishini) kunalokho obukudinga ngenxa yokuthi kwakungekho ukudla okwanele?**	1 = Rarely (one to two times in the past four weeks)<br>2 = Sometimes (three to 10 times in the past four weeks)<br>3 = Often (more than 10 times in the past four weeks)

FS5.6	In the past four weeks, did you have to eat fewer meals in a day because there was not enough food?<br><br>**Kulamasonto amane adlule, ingabe wena kwaphoqa ukuthi udle ukudla okuncane (ngokwezikhathi zokudla) ngosuku ngenxa yokuthi kwakungekho ukudla okwanele?**	1 = Rarely (one to two times in the past four weeks)
		2 = Sometimes (three to 10 times in the past four weeks)
		3 = Often (more than 10 times in the past four weeks)
FS5.7	In the past four weeks, was there ever no food to eat of any kind in your household because of lack of resources to get food?<br><br>**Kulamasonto amane adlule, kuke kwangabakhona noma ikuphi ukudla ekhaya ngenxa yokungabi nezinsiza zokuthola ukudla?**	1 = Rarely (one to two times in the past four weeks)
		2 = Sometimes (three to 10 times in the past four weeks)
		3 = Often (more than 10 times in the past four weeks)
FS5.8	In the past four weeks, did you go to sleep at night hungry because there was not enough food?<br><br>**Kulamasonto amane adlule, ingabe wena uye walala ebusuku ulambile ngenxa yokuthi bekungekho ukudla?**	1 = Rarely (one to two times in the past four weeks)
		2 = Sometimes (three to 10 times in the past four weeks)
		3 = Often (more than 10 times in the past four weeks)
FS5.9	In the past four weeks, did you go a whole day and night without eating anything because there was not enough food?<br><br>**Kulamasonto amane adlule, ngabe wena uye waqeda usuku lonke kanye nobusuku ungadla lutho ngenxa yokuthi bekungekho ukudla okwanele?**	1 = Rarely (one to two times in the past four weeks)
		2 = Sometimes (three to 10 times in the past four weeks)
		3 = Often (more than 10 times in the past four weeks)

Section 6: Plans

To be asked of all mothers regardless of which field visit this is<br><br>Kuzobuzwa kubobonke omama , noma ingabe ukuphi ukuvakashela			
P6.1	Have you gone back to work?<br>**Ingabe usubuyelile emsebenzini?**	1= Yes<br>➔ ***Skip to 6.3***	0= No
P6.2	Have you done any paid work since the baby was born?<br>**Ingabe usuke wasebenza umsebenzi okhokhelayo selokhu umntwana wazalwa?**	1= Yes	0= No<br>➔ ***Skip to 6.5***
P6.3	Is it the same job you did before the baby was born?<br>**Ingabe kusayiwo lomsebenzi obuwusebenza ngaphambili ungakabelethi?**	1= Yes<br>➔ ***Skip to 6.9***	0= No
P6.4	Are you working inside your home or outside your home?<br>**Ingabe usebenzela ngaphakathi ekhaya noma ngaphandle kwasekhaya**	1= Inside	2= outside<br>➔ ***Skip to 6.9***
P6.5	Are you still planning to go back to your previous work?<br><br>**Ingabe ngokohohlelo usazobuyela emsebenzini?**	1 = Yes	0 = No<br><br>➔ ***End interview***
P6.6	When are you planning to go back to work?<br><br>**Uhlele ukubuyela nini emsebenzini?**		
P6.7	Who will care for your baby when you go back to work?<br><br>**Ingabe ubani ozonakekela umntwana wakho ngenkathi usubuyele emsebenzini?**	1 = Childs grandmother	
		2 = Childs father	
		3 =Childs sibling	
		4 = Other relative	
		5 = Non relative or crèche	
		6 = Yourself (take child to work/work at home)	
P6.8	How will you feed your baby when you go back to work?<br><br>**Ingabe uzomupha kanjani umntwana wakho uma usubuyele emsebenzini?**	1 = Breastfeed<br><br>**Ubisi lwebele lodwa** ➔ ***End interview***	
		2 = Formula feed<br><br>**Ubisi lwefomula/ithini** ➔ ***End interview***	
		3 = Mixed feed<br><br>**Xuba ubisi lwebele nokunye engakaqedi isithupha sezinyanga** ➔ ***End interview***	

P6.9	Who is taking care of your baby while you at work?<br>**Ingabe ubani onakekela ingane yakho uma usemsebenzini?**	1 = Childs grandmother
		2 = Childs father
		3 =Childs sibling
		4 = Other relative
		5 = Non relative or crèche
		6 = Yourself (take child to work/work at home)
P6.10	How are you feeding your baby?<br>**Ingabe umupha kanjani umntwana wakho?**	1 = Breastfeed<br>**Ubisi lwebele lodwa**
		2 = Formula feed<br>**Ubisi lwefomula/ithini**
		3 = Mixed feed<br>**Xuba ubisi lwebele nokunye engakaqedi isithupha sezinyanga**
