# Supplementary material for: ‘I decided to go back to work so I can afford to buy her formula’: a longitudinal mixed-methods study to explore how women in informal work balance the competing demands of infant feeding and working to provide for their family
Source: BMC Public Health. 2020 Dec 2;20:1847. doi: 10.1186/s12889-020-09917-6 (PMC7709310; doi:10.1186/s12889-020-09917-6)
Supplement: Supplementary file 3 — Additional file 3. The Livelihood and Nurturing Care study (LiNCs): IDI interview guide plans for feeding and child care (pregnancy). [file 12889_2020_9917_MOESM3_ESM.pdf]

The Livelihood and Nurturing Care study (LiNCs): IDI interview guide plans for feeding and child care (pregnancy)

<u>Instructions to facilitators</u>

- Always use two audio recorders
- Before you start the interview speak into the recorder and provide the following information
  - Mother number
  - Date
  - Interviewers name
  - Interview name (pregnancy interview)
- **NOTE:** if the mother has scored > 13 on the PNDS include the optional question on anxiety and depression. Inform the LiNCs support team and **REFER** the mother to the clinic.

Introduction to the mother

Thank you for agreeing to participate in this interview. We have now switched on the audio-recorders. The reason for asking these questions is to understand your experiences of being pregnant while being an informal worker, and what your plans are about how to care for and feed your baby when he/she is born. If during the interview you do not want to answer one of the questions or you would like to stop, you are free to ask me to move to the next question or stop the interview at any time.

Interview Questions:

1. **Describe the work that you do**

   Probes: describe work type eg employed/own account/ employer in an informal business

   Describe what you do each working day from leaving home to returning home

   Describe the number of hours and days that you work

   Describe your relationship with your employer and with other workers in the area, your colleagues or co-workers?

   Describe the place where you are working? Describe amenities (water, toilets etc) that are available.

2. **Describe how you feel about being pregnant- describe your expectations regarding this pregnancy?**

   Probes: Is there anything that you worry about when you think of the birth of this baby?

3. **How has being pregnant affected the work that you do?**

   Probe: has the type of work, working hours or workload changed since you became pregnant?

Describe how your co-workers and your employer have responded to you being pregnant?

Describe whether being pregnant has affected your income in any way

Describe whether being pregnant has affected your health in any way

4. **Describe any people who have given you support since you have been pregnant and how they have supported you**

   Probe: describe support have you received from family members? Friends? Colleagues/co-workers? Community health workers? Clinic staff?

   Describe how the father has responded to the pregnancy and any support that he has provided

5. **Describe your experiences of receiving health care during this pregnancy?**

   Probes: Are you able to attend ANC? If yes, how do you manage working and going to clinic visits?

   How have you experienced attending clinic visits?

   Describe whether being an informal worker affects your experiences at the clinic and if so how

   Apart from the clinic, describe any other health services you have attended during this pregnancy? (private doctors, traditional healers or faith healers etc)

6. **Describe how you plan to care for and feed your baby, and how you will manage your work responsibilities once the baby is born**

   Probes: How much time do you plan to take off work when the baby is born?

   How are you going to financially support yourself while you are not working?

   How do you plan to feed the baby after birth?

   Describe how do you plan to care for your baby when you return to work? Who is going to care for the baby?

   Is there anything in particular that you expect to be a challenge when this baby is born?

   If employed- have you discussed your plans for the baby's birth with your employer? If so, describe how your employer responded.
