# Supplementary material for: ‘I decided to go back to work so I can afford to buy her formula’: a longitudinal mixed-methods study to explore how women in informal work balance the competing demands of infant feeding and working to provide for their family
Source: BMC Public Health. 2020 Dec 2;20:1847. doi: 10.1186/s12889-020-09917-6 (PMC7709310; doi:10.1186/s12889-020-09917-6)
Supplement: Supplementary file 4 — Additional file 4. The Livelihood and Nurturing Care study (LiNCs): IDI interview guide plans for feeding and child care (post-delivery). [file 12889_2020_9917_MOESM4_ESM.pdf]

The Livelihood and Nurturing Care study (LiNCs): IDI interview guide plans for feeding and child care (post-delivery)

Instructions to facilitators

- Always use two audio recorders
- Before you start the interview speak into the recorder and provide the following information
    - Mother number
    - Date
    - Interviewers name
    - Interview name (post-delivery interview *before returning to work*)
- NOTE: if the mother has scored > 13 on the PNDS include the optional question on anxiety and depression. Inform the LiNCs support team and REFER the mother to the clinic.

Introduction

Thank you for agreeing to participate in this interview. We have now switched on the audio-recorders. The reason for asking these questions is to understand your experiences of being having a baby while being an informal worker, and what your plans are about how to care for and feed your baby when you go back to work. If during the interview you do not want to answer one of the questions or you would like to stop, you are free to ask me to move to the next question or stop the interview at any time.

Interview questions:

1. **How are things going with your baby?**

    Probes: Experiences of caring for the baby, did everything go as you expected?

    Have you experienced any challenges in caring for your baby?

    Is there anything in particular that is worrying you about how things are going in caring for your baby?

2. **Can you tell me how you have experienced feeding overall since your baby was born and how you are feeding your baby now?**

    Probes: What is the reason why you made the particular feeding choice? Explain whether your work plans affect the feeding choice that you made in any way?

    Are you feeding your baby as planned, or did the plan change? If so, why did you change your planned feeding method?

    (If mothers are breastfeeding) are you also giving other food or fluids? Describe what other food/ fluids you have been giving to the baby and explain the reasons for this?

3. **Describe any people who have given you support since the baby was born and how they have supported you**

   Probe: describe support have you received from family members? Friends? Colleagues/co-workers? Community health workers? Clinic staff?

   Describe how the father has responded to the birth of the baby and any support that he has provided

4. **Describe how you plan to care for and feed your baby, as well as how you will manage your work responsibilities when you go back to work**

   Probes: How long will you take time take off work?

   How are you going to feed the baby when you go back to work? Would you consider expressing breastmilk to give to the baby while you are away from him/her?

   Describe how do you plan to care for your baby when you return to work? Who is going to care for the baby?

   Is there anything in particular that you expect to be a challenge when you go back to work? Is there anything that you worry about when you think of the future?

   Is there anything that is particularly worrying you about the future?

5. **How are you currently financially supporting yourself and the baby?**

   Probes: Do you another source of income while you are at home? Role of family members and father of child in supporting mother and childcare? Role of employers in supporting mother and childcare?

6. **Is there anything that is making you feel anxious or unhappy?**
