# Supplementary material for: ‘I decided to go back to work so I can afford to buy her formula’: a longitudinal mixed-methods study to explore how women in informal work balance the competing demands of infant feeding and working to provide for their family
Source: BMC Public Health. 2020 Dec 2;20:1847. doi: 10.1186/s12889-020-09917-6 (PMC7709310; doi:10.1186/s12889-020-09917-6)
Supplement: Supplementary file 5 — Additional file 5. The Livelihood and Nurturing Care study (LiNCs): IDI interview guide plans for return to work. [file 12889_2020_9917_MOESM5_ESM.docx]

**Instructions to facilitators**

- Always use two audio recorders
- Before you start the interview speak into the recorder and provide the following information
  - Mother number
  - Date
  - Interviewers name
  - Interview name (post-delivery interview)
- **NOTE:** if the mother has scored > 13 on the PNDS include the optional question on anxiety and depression. Inform the LiNCs support team and **REFER** the mother to the clinic.

**Introduction**

Thank you for agreeing to participate in this interview. We have now switched on the audio-recorders. The reason for asking these questions is to understand your experiences of returning to work as an informal worker with a young baby, and how you are caring for and feeding your baby while you are at work. If during the interview you do not want to answer one of the questions or you would like to stop, you are free to ask me to move to the next question or stop the interview at any time.

***Return to work***

1. **Describe what happened on the first day when you returned to work and how you felt on that day**
2. **Why did you chose to return to work at this time?**

**Probe:** How old was the baby when you went back to work

Describe the main reasons and what motivated you the most to go back to go back to work vs staying home for longer?

Describe whether anyone influenced you in any way when you were making the decision and whether this changed the decision you made to go back to work.

1. **Describe your experiences since you have been going back to work, particularly how things have changed *at work* since you have a new baby?**

Explain whether being a mother has changed the work that you do? Describe whether you are working the same hours as before, and whether the workload has changed in any way.

Do you receive the same payment for your work?

Does anyone help you with your work? Explain who helps you and how do they help you.

***Childcare***

1. **Where is your baby taken care of while you are at work?**

**Probe:** why did you chose this childcare option? What other alternatives for childcare did you consider? What things did you think about while you were making the decision?

Explain whether there was anyone who influenced your childcare decision? In what way did these people affect your decision?

Who is responsible for the care of the child when you are at work?

How do you feel about the current childcare arrangements? Do you have any concerns related to your current childcare?

Do you pay for childcare?

1. **Describe how you feed the baby now?**

**Probe:** How is the baby fed while you are at work?

How do you feed while you are at home?

Has your feeding changed since going back to work, and if so, how has it changed?

**IF THE MOTHER TAKES THE CHILD TO WORK WITH HER:**

1. **How do you feel about bringing your baby to work?**

How does the work environment affect you when you are caring for your baby? Describe any challenges that you have with taking your baby to work

How do you manage to do your work and care for your baby?

Describe who supports you during the day when you have the baby with you?

Describe how your colleagues have responded to you bringing your child to work

*If breastfeeding in the workplace:* how do you feel about breastfeeding while at work?

1. **Is there anything that is making you feel anxious or unhappy?**
